# Two Cases of Gastric Ulcer Treated by Peptonised Enemata

**Published:** 1884-03

**Authors:** 

**Affiliations:** Physicians to Bristol Royal Infirmary; Physicians to Bristol Royal Infirmary


### Clinical Records.

---

### TWO CASES OF GASTRIC ULCER TREATED BY PEPTONISED ENEMATA. By Drs. SPENCER and SHAW, Physicians to Bristol Royal Infirmary.

A. C., æt. 20, dressmaker. Admitted to Bristol Royal Infirmary, under Dr. Spencer, on April 9th, 1883. Had been under medical care for previous two or three years on account of anæmia and palpitation. Pain and tenderness at the epigastrium, made worse by food, and sickness for three months; has had frequent fainting fits. Three days before admission vomited a teacupful of dark-brown blood. On admission, extremely anæmic and weak; much pain, especially after food, and tenderness at epigastrium; no appetite; systolic pulmonic murmur; pulse 120, feeble; temperature normal. April 10th: Bowels opened by simple enema; motion very dark. April 11th: Vomited about 6 oz. of blood and clot; this repeated to same amount later in the day; motions quite black. April 13th: Nutrient enemata commenced to-day; to have no food or medicine by mouth. Each enema consisted of—Brandy, half an oz.; half an egg; Brand's essence of beef, two drachms; peptonised milk to 4 oz. An enema was given every four hours, and once in the twenty-four hours the bowel was washed out with luke-warm water. Two days later, on account of thirst, was allowed a very little peptonised milk to sip. No change was made in this treatment until April 27th (the 15th day). Then, there having been no vomiting, pain or hæmorrhage, nor discomfort of any kind, and the patient having improved greatly in appearance, the enemas were discontinued, and the patient was fed by the mouth with peptonised milk. In two days ordinary milk and lime water was substituted; then beef-tea was added. On May 2nd chicken was allowed. May 7th: There had been no symptoms, and patient felt quite well; was then put upon pills of sulphate of iron and carbonate of potash. May 9th: Patient got up. May 16th: Patient left the Infirmary. She continued to take the pills, as an out-patient, for some time, and finally was completely cured of the anæmia. In January, 1884, the patient was maintaining her health, was of good colour, and said that she had never felt so well in her life.

F. S., æt. 26, domestic servant. Admitted under Dr. Shaw. Had suffered from indigestion for a year, more severely for the last six months, but had never vomited. On December 14th, 1883, while walking in the street, became suddenly faint, and then vomited a large quantity of blood and food; was taken to Infirmary, where on arriving she again vomited, a pint of "coffee-ground" vomit being brought up. Was given a hypodermic injection of ergotine, and placed on diet of milk and beef-tea. Heart, lungs and urine revealed no abnormality. December 15th: Ordered bismuth mixture. Two attacks of hæmatemesis, amounting to two pints, preceded by gnawing pain in epigastrium. December 16th: Stomach rather distended; no tenderness on palpation. Tongue, white fur. Pulse sharp and collapsing. Tarry motion passed after simple enema. No vomiting. December 17th: No vomiting; no pain or tenderness in epigastrium. Ordered bismuth and morphia in powder and ice to suck. December 18th: Tarry motion after enema. Two attacks of hæmatemesis—22 oz., preceded by feeling of faintness, but not by pain. Ordered Pulv. Acid. Tannic. gr. x. quartis horis sum.; nothing else of any kind whatever to be taken by the mouth, but every four hours enemata, consisting alternately of 4 oz. of peptonised milk and of the peptonised solution of beef, to be administered regularly day and night. Patient excessively blanched. December 19th: Well able to retain enemata; bowels not moved. Vomits after Tannic acid. Complains of extreme thirst, but no pain. Pulse sharp and jerky; well-marked bruit in pulmonary area. December 20th: Half-doses of Tannic acid vomited, but without trace of blood. Bowels not moved. Free from all pain. Allowed a little iced water by mouth, and ordered Pil. Opii (gr. ½) ter die. December 23rd: Bowels still unmoved. Ordered twice daily one of Slinger's nutrient suppositories; treatment otherwise unaltered. December 24th: Bowels moved during night; motion light-coloured and loose. No vomiting. Some pain, but no tenderness in epigastrium. December 27th: Vomited twice yesterday; thin fluid, without trace of blood; neither nausea nor pain before vomiting. Bowels moved; motion light and loose. Still very thirsty. Tongue dry. Nutrient enemata increased to ℥ vj. each, as they are retained without difficulty. December 28th: Vomited twice. Pale watery motion passed, showing no sign of blood. Opium discontinued yesterday. December 31st: For last two days there has been diarrhœa, with griping pains in bowels; motions very offensive. Ordered to take creasote in pill as well as opium, which was recommenced. January 2nd, 1884: Pain and diarrhœa relieved; bowels moved once to-day. Tongue dry, with brownish fur. No difficulty in retaining enemata. January 7th: Vomiting continues absent. A light-coloured motion passed on the 5th. Tongue moister, and patient begins to feel hungry. January 8th: A light loose motion passed to-day after three days' confinement. To-day patient is allowed to have the peptonised milk by the mouth, having taken no nutriment by that method for twenty-one days. Enemata to be continued every four hours as before, the peptonised solution of beef alone being used for that purpose. During the preceding three weeks patient has gained strength considerably, *probably* lost weight to some extent, and not perceptibly improved in respect of the deficiency of hæmoglobin caused by the hæmatemesis. January 11th: Peptonised milk by mouth increased to two pints. January 14th: One egg, beaten up, added to food by mouth; nutrient enemata continued as before. January 18th: Bowels are moved daily; motions rather dark and loose. Has had arrowroot in addition by mouth since 16th. Nutrient enemata four times in the twenty-four hours. January 21st: Patient lifted out of bed and weighed; weight, 7st. 3lb. January 22nd: Milk cake added to diet. January 24th: Has no epigastric pain now. Pills of opium and creasote discontinued, and pill of aloes and iron given instead. Nutrient enemata to be left off to-day, having been employed continuously for thirty-seven days. Throughout patient retained them easily, and suffered no inconvenience from them. January 28th: Weight, 7st. 6lb. Bread added to diet, and beef-tea given instead of the peptonised milk. January 31st: Bowels confined; pain in chest. Aloetic pill increased in strength. Fish added to diet. February 4th: Weight, 7st 11lb. Bowels now moved daily. Patient allowed to get up for an hour to-day. February 11th: Weight, 8st 3lb. Gets up for three hours daily; has some giddiness on first rising. To have meat finely minced for dinner. February 12th: Hæmoglobin estimated at 60 per cent. Corpuscles at 3,960,000 per cubic millimetre. February 18th: Weight, 8st. 7lb. Is up all day now. Constant slight pain in chest not influenced by food; not conscious of any other discomfort. Has not vomited since December 28th. Will be discharged in a day or two.

This case is a good illustration of the efficiency of peptonised enemata to maintain life for a prolonged period, without any assistance from digestion by the stomach and small intestine. It also shows the great value of rest to the stomach in the treatment of gastric ulcer. Notwithstanding the alarming hæmorrhage from the stomach, none such occurred again when, by no longer putting digestible substances into the organ, the fluxionary hyperæmia of digestion was no more induced. That the powers of digestion were increased by rest rather than diminished by disuse is shown by the rapid gain in weight when a moderately liberal diet permitted its occurrence.

